# 3,4-Bis[4-(4-meth­oxy­phen­oxy)phen­yl]-1-methyl-1*H*-pyrrole-2,5-dione

**DOI:** 10.1107/S1600536811032594

**Published:** 2011-08-17

**Authors:** Weili Ma, Gang Chen, Wenjing Wang, Zhenting Du

**Affiliations:** a College of Science, Northwest A&F University, Yangling, Shaanxi, 712100, People’s Republic of China

## Abstract

The title compound, C_31_H_25_NO_6_, has a structure related to other 3,4-diaryl-substituted maleic anhydride derivatives which have been shown to be useful as photochromic materials. The dihedral angles between the maleimide ring system and the benzene rings bonded to it are 44.48 (3) and 17.89 (3)°, while the angles between each of the latter rings and the corresponding ether bridging connected meth­oxy­benzene rings are 78.61 (8) and 72.67 (7)°. In the crystal, the molecules are linked by C—H⋯O inter­actions.

## Related literature

For background to the use of 3,4-diaryl-substituted maleic anhydride derivatives, see: Yeh *et al.* (2003 ▶); Franc *et al.* (2007 ▶).
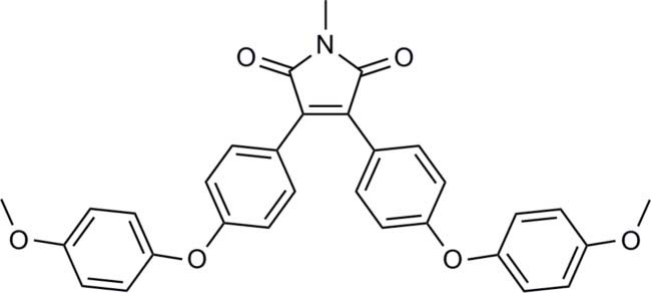


## Experimental

### 

#### Crystal data

C_31_H_25_NO_6_
*M* *_r_* = 507.52
Triclinic, 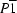

*a* = 8.751 (4) Å
*b* = 10.681 (5) Å
*c* = 13.630 (7) Å
α = 97.956 (5)°
β = 91.951 (4)°
γ = 93.615 (5)°
*V* = 1258.1 (11) Å^3^
*Z* = 2
Mo *K*α radiation
μ = 0.09 mm^−1^
*T* = 296 K
0.32 × 0.26 × 0.24 mm

#### Data collection

Bruker APEXII CCD diffractometer
Absorption correction: multi-scan (*SADABS*; Bruker, 2009 ▶) *T*_min_ = 0.971, *T*_max_ = 0.978
6956 measured reflections
4439 independent reflections
2861 reflections with *I* > 2σ(*I*)
*R*_int_ = 0.028

#### Refinement

*R*[*F* ^2^ > 2σ(*F* ^2^)] = 0.050
*wR*(*F* ^2^) = 0.132
*S* = 1.04
4439 reflections
347 parameters
H-atom parameters constrained
Δρ_max_ = 0.17 e Å^−3^
Δρ_min_ = −0.19 e Å^−3^

### 

Data collection: *APEX2* (Bruker, 2009 ▶); cell refinement: *SAINT* (Bruker, 2009 ▶); data reduction: *SAINT*; program(s) used to solve structure: *SHELXS97* (Sheldrick, 2008 ▶); program(s) used to refine structure: *SHELXL97* (Sheldrick, 2008 ▶); molecular graphics: *SHELXTL* (Sheldrick, 2008 ▶); software used to prepare material for publication: *SHELXTL*.

## Supplementary Material

Crystal structure: contains datablock(s) I, global. DOI: 10.1107/S1600536811032594/lr2020sup1.cif

Structure factors: contains datablock(s) I. DOI: 10.1107/S1600536811032594/lr2020Isup2.hkl

Supplementary material file. DOI: 10.1107/S1600536811032594/lr2020Isup3.cml

Additional supplementary materials:  crystallographic information; 3D view; checkCIF report

## Figures and Tables

**Table 1:** Hydrogen-bond geometry (Å, °)

*D*—H⋯*A*	*D*—H	H⋯*A*	*D*⋯*A*	*D*—H⋯*A*
C4—H4⋯O4^i^	0.93	2.43	3.294 (3)	155
C18—H18*B*⋯O3^ii^	0.96	2.37	3.325 (3)	171

Symmetry codes: (i) 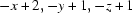; (ii) 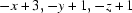.

**Fig. 1.**
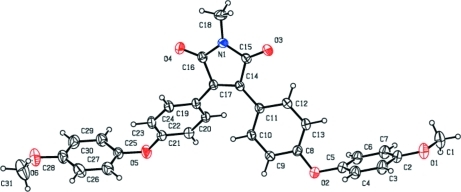
The molecular structure of (I). Displacement ellipsoids are drawn at the 30% probability level.

## supplementary crystallographic information

### Comment

3,4-Diaryl substituted maleic anhydride is a conjugated unit which has interesting optical and electronic properties. A number of 3,4-Diaryl substituted maleic anhydride derivatives have been designed and synthesized to be used as photochromic materials (Franc *et al.*, 2007, Yeh *et al.*, 2003). In the course of exploring new potential photochromic compounds, we obtained the title compound. The synthesis was accomplished through a key palladium catalysed C—O bond formation.

The molecule holds two long-chain branches with methyl group at the end to enhance its solubility. The dihedral angles between the the maleimide five-membered ring and the benzene rings (C8-C13) and ( C18-C23 ) directly bonded to it are of 17.89 (3)° and 44.48 (3)° respectively. On the other hand, the dihedral angles between each of the latter rings and the corresponding ether bridging connected methoxybenzene ring,  (C2-C7 ) and (C25-C30) respectively, are of  78.61 (8) and 72.67 (7) °. The crystal packing is stabilized by C—H ···O interactions.

### Experimental

According to the works of Yeh (Yeh *et al.* 2003), 3,4-bis(4-bromophenyl)-1-methyl-1*H*-pyrrole-2,5-dione (0.41 g, 1 mmol), 4-methoxyphenol (0.372 g, 3 mmol), Pd(PPh_3_)_4_ (0.115 g, 0.1 mmol), *t*-BuONa (0.3 g, 3 mmol), P(*t*-Bu)_3_^.^HBF_4_ (58 mg, 0.2 mmol) were refluxed in anhydrous THF (40 ml), under argon, for 6 h. Afterwards, the reaction mixture was extracted with dichloro methane (40 ml×3). The organic layers were combined, washed with brine and the solvent evaporated. The residue was purified through column chromotography to give the title compound. The product was dissolved in methanol and red block crystals were formed by slow evaporation at room temperature over one week.

### Refinement

All H atoms were placed in idealized positions (C—H = 0.93–0.97 Å) and refined as riding atoms with *U*_iso_(H) = 1.2*U*_eq_(C_aromatic_) and with *U*_iso_(H) = 1.5*U*_eq_(C_methyl_).

### Crystal data

**Table:**

C_31_H_25_NO_6_	*Z* = 2
*M**_r_* = 507.52	*F*(000) = 532
Triclinic, *P*1	*D*_x_ = 1.340 Mg m^−^^3^
Hall symbol: -P 1	Melting point: 373 K
*a* = 8.751 (4) Å	Mo *K*α radiation, λ = 0.71073 Å
*b* = 10.681 (5) Å	Cell parameters from 1817 reflections
*c* = 13.630 (7) Å	θ = 2.3–22.8°
α = 97.956 (5)°	µ = 0.09 mm^−^^1^
β = 91.951 (4)°	*T* = 296 K
γ = 93.615 (5)°	Block, red
*V* = 1258.1 (11) Å^3^	0.32 × 0.26 × 0.24 mm

### Data collection

**Table:**

Bruker APEXII CCD diffractometer	4439 independent reflections
Radiation source: fine-focus sealed tube	2861 reflections with *I* > 2σ(*I*)
graphite	*R*_int_ = 0.028
φ and ω scans	θ_max_ = 25.2°, θ_min_ = 2.3°
Absorption correction: multi-scan (*SADABS*; Bruker, 2009)	*h* = −8→10
*T*_min_ = 0.971, *T*_max_ = 0.978	*k* = −12→12
6956 measured reflections	*l* = −16→16

### Refinement

**Table:**

Refinement on *F*^2^	Secondary atom site location: difference Fourier map
Least-squares matrix: full	Hydrogen site location: inferred from neighbouring sites
*R*[*F*^2^ > 2σ(*F*^2^)] = 0.050	H-atom parameters constrained
*wR*(*F*^2^) = 0.132	*w* = 1/[σ^2^(*F*_o_^2^) + (0.0592*P*)^2^ + 0.0849*P*] where *P* = (*F*_o_^2^ + 2*F*_c_^2^)/3
*S* = 1.04	(Δ/σ)_max_ < 0.001
4439 reflections	Δρ_max_ = 0.17 e Å^−^^3^
347 parameters	Δρ_min_ = −0.19 e Å^−^^3^
0 restraints	Extinction correction: *SHELXL*, Fc^*^=kFc[1+0.001xFc^2^λ^3^/sin(2θ)]^-1/4^
Primary atom site location: structure-invariant direct methods	Extinction coefficient: 0.017 (2)

### Special details

**Table:**

Geometry. All e.s.d.'s (except the e.s.d. in the dihedral angle between two l.s. planes) are estimated using the full covariance matrix. The cell e.s.d.'s are taken into account individually in the estimation of e.s.d.'s in distances, angles and torsion angles; correlations between e.s.d.'s in cell parameters are only used when they are defined by crystal symmetry. An approximate (isotropic) treatment of cell e.s.d.'s is used for estimating e.s.d.'s involving l.s. planes.
Refinement. Refinement of *F*^2^ against ALL reflections. The weighted *R*-factor *wR* and goodness of fit *S* are based on *F*^2^, conventional *R*-factors *R* are based on *F*, with *F* set to zero for negative *F*^2^. The threshold expression of *F*^2^ > σ(*F*^2^) is used only for calculating *R*-factors(gt) *etc*. and is not relevant to the choice of reflections for refinement. *R*-factors based on *F*^2^ are statistically about twice as large as those based on *F*, and *R*- factors based on ALL data will be even larger.

### Fractional atomic coordinates and isotropic or equivalent isotropic displacement parameters (Å^2^)

**Table:**

	*x*	*y*	*z*	*U*_iso_*/*U*_eq_	
O3	1.34578 (18)	0.43301 (16)	0.57789 (11)	0.0535 (5)	
O2	0.71603 (19)	0.58407 (17)	0.79815 (11)	0.0609 (5)	
C11	0.9988 (2)	0.3937 (2)	0.59643 (14)	0.0372 (5)	
N1	1.3183 (2)	0.27986 (17)	0.44303 (13)	0.0438 (5)	
O4	1.21810 (18)	0.11724 (15)	0.32755 (11)	0.0519 (4)	
C19	0.9188 (2)	0.1458 (2)	0.42139 (14)	0.0382 (5)	
C15	1.2649 (3)	0.3562 (2)	0.52269 (16)	0.0403 (5)	
O5	0.54088 (19)	−0.10824 (17)	0.34147 (11)	0.0638 (5)	
C16	1.2023 (3)	0.1979 (2)	0.39736 (16)	0.0410 (6)	
C14	1.0950 (2)	0.3234 (2)	0.52514 (14)	0.0372 (5)	
C8	0.8148 (3)	0.5244 (2)	0.73415 (16)	0.0446 (6)	
C22	0.6666 (3)	−0.0239 (2)	0.36263 (16)	0.0443 (6)	
C20	0.8297 (3)	0.0946 (2)	0.49091 (15)	0.0402 (5)	
H20	0.8556	0.1168	0.5581	0.048*	
C21	0.7048 (3)	0.0122 (2)	0.46208 (16)	0.0432 (6)	
H21	0.6457	−0.0195	0.5096	0.052*	
C10	0.8407 (3)	0.3901 (2)	0.58153 (16)	0.0431 (6)	
H10	0.7953	0.3436	0.5241	0.052*	
C17	1.0589 (2)	0.2265 (2)	0.45106 (14)	0.0372 (5)	
O1	0.9801 (2)	0.87749 (17)	1.13293 (12)	0.0677 (5)	
C12	1.0617 (3)	0.4688 (2)	0.68122 (16)	0.0488 (6)	
H12	1.1675	0.4751	0.6926	0.059*	
C2	0.9115 (3)	0.7981 (2)	1.05445 (16)	0.0497 (6)	
C24	0.8780 (3)	0.1087 (2)	0.32176 (15)	0.0490 (6)	
H24	0.9361	0.1410	0.2739	0.059*	
C9	0.7493 (3)	0.4533 (2)	0.64913 (16)	0.0459 (6)	
H9	0.6436	0.4482	0.6376	0.055*	
C5	0.7808 (3)	0.6555 (2)	0.88531 (16)	0.0504 (6)	
C13	0.9718 (3)	0.5342 (2)	0.74917 (17)	0.0521 (6)	
H13	1.0171	0.5848	0.8049	0.063*	
C23	0.7526 (3)	0.0248 (2)	0.29221 (16)	0.0525 (7)	
H23	0.7266	0.0015	0.2252	0.063*	
C25	0.5045 (3)	−0.1585 (3)	0.24244 (17)	0.0515 (6)	
C6	0.8242 (3)	0.5976 (2)	0.96397 (18)	0.0576 (7)	
H6	0.8092	0.5101	0.9604	0.069*	
C28	0.4252 (3)	−0.2663 (3)	0.05195 (19)	0.0608 (7)	
C18	1.4766 (3)	0.2811 (3)	0.41449 (19)	0.0626 (7)	
H18A	1.5341	0.2340	0.4562	0.094*	
H18B	1.5196	0.3670	0.4217	0.094*	
H18C	1.4808	0.2432	0.3466	0.094*	
C30	0.5342 (3)	−0.2811 (2)	0.21135 (19)	0.0594 (7)	
H30	0.5814	−0.3283	0.2547	0.071*	
O6	0.3898 (3)	−0.3287 (2)	−0.04174 (14)	0.0908 (7)	
C7	0.8906 (3)	0.6690 (2)	1.04909 (17)	0.0562 (7)	
H7	0.9209	0.6294	1.1025	0.067*	
C3	0.8626 (3)	0.8553 (3)	0.97553 (17)	0.0613 (7)	
H3	0.8733	0.9431	0.9796	0.074*	
C4	0.7982 (3)	0.7842 (3)	0.89093 (18)	0.0595 (7)	
H4	0.7665	0.8236	0.8377	0.071*	
C29	0.4940 (3)	−0.3346 (3)	0.1158 (2)	0.0663 (8)	
H29	0.5140	−0.4183	0.0945	0.080*	
C26	0.4343 (3)	−0.0889 (3)	0.1802 (2)	0.0709 (8)	
H26	0.4131	−0.0058	0.2025	0.085*	
C27	0.3944 (3)	−0.1425 (3)	0.0832 (2)	0.0769 (9)	
H27	0.3473	−0.0954	0.0398	0.092*	
C31	0.3351 (5)	−0.2593 (4)	−0.1141 (2)	0.1234 (15)	
H31A	0.2410	−0.2241	−0.0937	0.185*	
H31B	0.3172	−0.3141	−0.1759	0.185*	
H31C	0.4096	−0.1919	−0.1220	0.185*	
C1	1.0342 (4)	0.8222 (3)	1.21523 (18)	0.0813 (10)	
H1A	0.9499	0.7790	1.2427	0.122*	
H1B	1.0809	0.8873	1.2647	0.122*	
H1C	1.1084	0.7628	1.1939	0.122*	

### Atomic displacement parameters (Å^2^)

**Table:**

	*U*^11^	*U*^22^	*U*^33^	*U*^12^	*U*^13^	*U*^23^
O3	0.0445 (10)	0.0525 (11)	0.0574 (10)	−0.0065 (8)	−0.0083 (8)	−0.0066 (8)
O2	0.0527 (11)	0.0727 (13)	0.0510 (10)	0.0111 (9)	−0.0025 (8)	−0.0160 (9)
C11	0.0393 (13)	0.0336 (12)	0.0375 (12)	0.0001 (10)	−0.0038 (10)	0.0028 (10)
N1	0.0360 (11)	0.0419 (12)	0.0510 (11)	0.0021 (9)	−0.0010 (9)	−0.0016 (9)
O4	0.0580 (11)	0.0497 (11)	0.0443 (9)	0.0024 (8)	0.0017 (8)	−0.0061 (8)
C19	0.0442 (13)	0.0342 (13)	0.0342 (12)	0.0009 (10)	−0.0060 (10)	0.0008 (10)
C15	0.0398 (13)	0.0348 (13)	0.0447 (13)	0.0017 (11)	−0.0079 (11)	0.0034 (11)
O5	0.0578 (11)	0.0715 (13)	0.0529 (10)	−0.0240 (10)	0.0025 (9)	−0.0109 (9)
C16	0.0473 (14)	0.0367 (13)	0.0387 (12)	0.0028 (11)	−0.0050 (11)	0.0059 (11)
C14	0.0403 (13)	0.0369 (13)	0.0347 (11)	0.0019 (10)	−0.0061 (10)	0.0076 (10)
C8	0.0501 (15)	0.0417 (14)	0.0405 (13)	0.0072 (11)	0.0004 (11)	−0.0003 (11)
C22	0.0417 (14)	0.0430 (14)	0.0449 (13)	−0.0003 (11)	−0.0017 (11)	−0.0031 (11)
C20	0.0471 (14)	0.0391 (13)	0.0329 (11)	0.0051 (11)	−0.0050 (10)	0.0010 (10)
C21	0.0476 (14)	0.0407 (14)	0.0411 (13)	0.0009 (11)	0.0012 (11)	0.0065 (10)
C10	0.0457 (14)	0.0403 (14)	0.0401 (12)	0.0031 (11)	−0.0086 (11)	−0.0030 (10)
C17	0.0409 (13)	0.0381 (13)	0.0319 (11)	0.0028 (10)	−0.0044 (10)	0.0037 (10)
O1	0.1001 (15)	0.0534 (11)	0.0455 (10)	−0.0008 (10)	−0.0102 (10)	−0.0008 (9)
C12	0.0394 (14)	0.0576 (16)	0.0457 (13)	0.0011 (12)	−0.0057 (11)	−0.0030 (12)
C2	0.0640 (17)	0.0467 (16)	0.0369 (13)	0.0044 (12)	0.0025 (12)	−0.0002 (11)
C24	0.0485 (15)	0.0596 (16)	0.0363 (13)	−0.0085 (12)	−0.0009 (11)	0.0040 (11)
C9	0.0407 (14)	0.0481 (15)	0.0464 (13)	0.0070 (11)	−0.0078 (11)	−0.0016 (11)
C5	0.0515 (15)	0.0553 (17)	0.0415 (14)	0.0076 (12)	0.0015 (11)	−0.0045 (12)
C13	0.0504 (16)	0.0555 (16)	0.0443 (13)	−0.0020 (12)	−0.0068 (12)	−0.0100 (12)
C23	0.0538 (16)	0.0639 (17)	0.0340 (12)	−0.0079 (13)	−0.0030 (11)	−0.0075 (12)
C25	0.0441 (15)	0.0543 (17)	0.0497 (14)	−0.0118 (12)	−0.0005 (12)	−0.0083 (13)
C6	0.0731 (18)	0.0424 (15)	0.0551 (15)	0.0045 (13)	0.0021 (13)	−0.0013 (13)
C28	0.0639 (18)	0.0569 (19)	0.0544 (16)	−0.0125 (14)	−0.0061 (14)	−0.0074 (14)
C18	0.0391 (15)	0.0632 (18)	0.0825 (19)	0.0018 (13)	0.0045 (13)	−0.0001 (15)
C30	0.0657 (18)	0.0500 (17)	0.0594 (16)	−0.0001 (14)	−0.0067 (13)	0.0012 (13)
O6	0.1134 (18)	0.0910 (16)	0.0576 (12)	−0.0200 (13)	−0.0130 (12)	−0.0100 (11)
C7	0.0745 (18)	0.0495 (17)	0.0461 (14)	0.0068 (13)	0.0010 (13)	0.0110 (12)
C3	0.087 (2)	0.0448 (16)	0.0504 (15)	0.0057 (14)	−0.0025 (14)	0.0031 (12)
C4	0.0746 (19)	0.0600 (19)	0.0451 (14)	0.0110 (14)	−0.0028 (13)	0.0094 (13)
C29	0.079 (2)	0.0475 (17)	0.0668 (18)	0.0008 (15)	−0.0003 (15)	−0.0087 (14)
C26	0.070 (2)	0.0564 (18)	0.079 (2)	0.0076 (15)	−0.0145 (16)	−0.0128 (16)
C27	0.075 (2)	0.076 (2)	0.076 (2)	0.0034 (17)	−0.0265 (16)	0.0060 (17)
C31	0.146 (4)	0.146 (4)	0.072 (2)	−0.032 (3)	−0.039 (2)	0.024 (2)
C1	0.119 (3)	0.072 (2)	0.0490 (16)	−0.0006 (18)	−0.0213 (17)	0.0008 (15)

### Geometric parameters (Å, °)

**Table:**

O3—C15	1.208 (2)	C24—H24	0.9300
O2—C8	1.372 (3)	C9—H9	0.9300
O2—C5	1.401 (3)	C5—C4	1.365 (4)
C11—C12	1.388 (3)	C5—C6	1.365 (3)
C11—C10	1.389 (3)	C13—H13	0.9300
C11—C14	1.467 (3)	C23—H23	0.9300
N1—C16	1.371 (3)	C25—C26	1.358 (4)
N1—C15	1.378 (3)	C25—C30	1.363 (4)
N1—C18	1.451 (3)	C6—C7	1.385 (3)
O4—C16	1.209 (2)	C6—H6	0.9300
C19—C24	1.389 (3)	C28—C29	1.359 (4)
C19—C20	1.394 (3)	C28—O6	1.372 (3)
C19—C17	1.465 (3)	C28—C27	1.378 (4)
C15—C14	1.508 (3)	C18—H18A	0.9600
O5—C22	1.374 (3)	C18—H18B	0.9600
O5—C25	1.400 (3)	C18—H18C	0.9600
C16—C17	1.501 (3)	C30—C29	1.373 (3)
C14—C17	1.356 (3)	C30—H30	0.9300
C8—C13	1.378 (3)	O6—C31	1.402 (4)
C8—C9	1.380 (3)	C7—H7	0.9300
C22—C23	1.377 (3)	C3—C4	1.372 (3)
C22—C21	1.380 (3)	C3—H3	0.9300
C20—C21	1.370 (3)	C4—H4	0.9300
C20—H20	0.9300	C29—H29	0.9300
C21—H21	0.9300	C26—C27	1.390 (3)
C10—C9	1.375 (3)	C26—H26	0.9300
C10—H10	0.9300	C27—H27	0.9300
O1—C2	1.366 (3)	C31—H31A	0.9600
O1—C1	1.419 (3)	C31—H31B	0.9600
C12—C13	1.380 (3)	C31—H31C	0.9600
C12—H12	0.9300	C1—H1A	0.9600
C2—C7	1.371 (3)	C1—H1B	0.9600
C2—C3	1.378 (3)	C1—H1C	0.9600
C24—C23	1.384 (3)		
			
C8—O2—C5	117.01 (18)	C8—C13—H13	120.1
C12—C11—C10	116.8 (2)	C12—C13—H13	120.1
C12—C11—C14	121.5 (2)	C22—C23—C24	119.6 (2)
C10—C11—C14	121.65 (19)	C22—C23—H23	120.2
C16—N1—C15	110.42 (19)	C24—C23—H23	120.2
C16—N1—C18	124.55 (19)	C26—C25—C30	120.8 (2)
C15—N1—C18	124.95 (19)	C26—C25—O5	120.8 (2)
C24—C19—C20	117.7 (2)	C30—C25—O5	118.4 (2)
C24—C19—C17	120.5 (2)	C5—C6—C7	120.1 (2)
C20—C19—C17	121.60 (18)	C5—C6—H6	120.0
O3—C15—N1	123.5 (2)	C7—C6—H6	120.0
O3—C15—C14	129.2 (2)	C29—C28—O6	115.8 (3)
N1—C15—C14	107.27 (18)	C29—C28—C27	120.0 (2)
C22—O5—C25	118.76 (18)	O6—C28—C27	124.2 (3)
O4—C16—N1	124.5 (2)	N1—C18—H18A	109.5
O4—C16—C17	128.1 (2)	N1—C18—H18B	109.5
N1—C16—C17	107.38 (19)	H18A—C18—H18B	109.5
C17—C14—C11	131.1 (2)	N1—C18—H18C	109.5
C17—C14—C15	107.03 (19)	H18A—C18—H18C	109.5
C11—C14—C15	121.86 (19)	H18B—C18—H18C	109.5
O2—C8—C13	124.0 (2)	C25—C30—C29	119.7 (3)
O2—C8—C9	116.4 (2)	C25—C30—H30	120.2
C13—C8—C9	119.6 (2)	C29—C30—H30	120.2
O5—C22—C23	124.4 (2)	C28—O6—C31	118.4 (3)
O5—C22—C21	115.7 (2)	C2—C7—C6	119.9 (2)
C23—C22—C21	119.9 (2)	C2—C7—H7	120.1
C21—C20—C19	121.22 (19)	C6—C7—H7	120.1
C21—C20—H20	119.4	C4—C3—C2	120.7 (2)
C19—C20—H20	119.4	C4—C3—H3	119.6
C20—C21—C22	120.2 (2)	C2—C3—H3	119.6
C20—C21—H21	119.9	C5—C4—C3	119.7 (2)
C22—C21—H21	119.9	C5—C4—H4	120.2
C9—C10—C11	122.0 (2)	C3—C4—H4	120.2
C9—C10—H10	119.0	C28—C29—C30	120.6 (3)
C11—C10—H10	119.0	C28—C29—H29	119.7
C14—C17—C19	133.4 (2)	C30—C29—H29	119.7
C14—C17—C16	107.81 (18)	C25—C26—C27	119.7 (3)
C19—C17—C16	118.61 (18)	C25—C26—H26	120.1
C2—O1—C1	117.5 (2)	C27—C26—H26	120.1
C13—C12—C11	121.9 (2)	C28—C27—C26	119.3 (3)
C13—C12—H12	119.1	C28—C27—H27	120.3
C11—C12—H12	119.1	C26—C27—H27	120.3
O1—C2—C7	125.0 (2)	O6—C31—H31A	109.5
O1—C2—C3	115.7 (2)	O6—C31—H31B	109.5
C7—C2—C3	119.3 (2)	H31A—C31—H31B	109.5
C23—C24—C19	121.3 (2)	O6—C31—H31C	109.5
C23—C24—H24	119.3	H31A—C31—H31C	109.5
C19—C24—H24	119.3	H31B—C31—H31C	109.5
C10—C9—C8	119.9 (2)	O1—C1—H1A	109.5
C10—C9—H9	120.1	O1—C1—H1B	109.5
C8—C9—H9	120.1	H1A—C1—H1B	109.5
C4—C5—C6	120.4 (2)	O1—C1—H1C	109.5
C4—C5—O2	119.0 (2)	H1A—C1—H1C	109.5
C6—C5—O2	120.6 (2)	H1B—C1—H1C	109.5
C8—C13—C12	119.8 (2)		
			
C16—N1—C15—O3	176.8 (2)	C14—C11—C12—C13	−179.3 (2)
C18—N1—C15—O3	−0.1 (3)	C1—O1—C2—C7	0.1 (4)
C16—N1—C15—C14	−2.9 (2)	C1—O1—C2—C3	−179.1 (2)
C18—N1—C15—C14	−179.8 (2)	C20—C19—C24—C23	0.6 (3)
C15—N1—C16—O4	−177.3 (2)	C17—C19—C24—C23	175.0 (2)
C18—N1—C16—O4	−0.4 (3)	C11—C10—C9—C8	0.9 (3)
C15—N1—C16—C17	1.7 (2)	O2—C8—C9—C10	−179.7 (2)
C18—N1—C16—C17	178.6 (2)	C13—C8—C9—C10	1.8 (3)
C12—C11—C14—C17	164.1 (2)	C8—O2—C5—C4	101.6 (3)
C10—C11—C14—C17	−17.0 (3)	C8—O2—C5—C6	−79.2 (3)
C12—C11—C14—C15	−18.0 (3)	O2—C8—C13—C12	178.9 (2)
C10—C11—C14—C15	160.9 (2)	C9—C8—C13—C12	−2.7 (4)
O3—C15—C14—C17	−176.6 (2)	C11—C12—C13—C8	0.9 (4)
N1—C15—C14—C17	3.1 (2)	O5—C22—C23—C24	−179.5 (2)
O3—C15—C14—C11	5.1 (3)	C21—C22—C23—C24	0.7 (4)
N1—C15—C14—C11	−175.27 (18)	C19—C24—C23—C22	−0.4 (4)
C5—O2—C8—C13	−2.2 (3)	C22—O5—C25—C26	−77.4 (3)
C5—O2—C8—C9	179.4 (2)	C22—O5—C25—C30	105.8 (3)
C25—O5—C22—C23	6.4 (4)	C4—C5—C6—C7	−2.0 (4)
C25—O5—C22—C21	−173.8 (2)	O2—C5—C6—C7	178.9 (2)
C24—C19—C20—C21	−1.0 (3)	C26—C25—C30—C29	0.6 (4)
C17—C19—C20—C21	−175.4 (2)	O5—C25—C30—C29	177.4 (2)
C19—C20—C21—C22	1.3 (3)	C29—C28—O6—C31	−172.7 (3)
O5—C22—C21—C20	179.1 (2)	C27—C28—O6—C31	7.8 (4)
C23—C22—C21—C20	−1.2 (4)	O1—C2—C7—C6	−177.7 (2)
C12—C11—C10—C9	−2.6 (3)	C3—C2—C7—C6	1.5 (4)
C14—C11—C10—C9	178.4 (2)	C5—C6—C7—C2	0.5 (4)
C11—C14—C17—C19	−8.7 (4)	O1—C2—C3—C4	177.1 (2)
C15—C14—C17—C19	173.2 (2)	C7—C2—C3—C4	−2.2 (4)
C11—C14—C17—C16	176.1 (2)	C6—C5—C4—C3	1.3 (4)
C15—C14—C17—C16	−2.0 (2)	O2—C5—C4—C3	−179.5 (2)
C24—C19—C17—C14	142.0 (2)	C2—C3—C4—C5	0.7 (4)
C20—C19—C17—C14	−43.8 (3)	O6—C28—C29—C30	−180.0 (2)
C24—C19—C17—C16	−43.2 (3)	C27—C28—C29—C30	−0.4 (4)
C20—C19—C17—C16	131.0 (2)	C25—C30—C29—C28	0.1 (4)
O4—C16—C17—C14	179.3 (2)	C30—C25—C26—C27	−1.0 (4)
N1—C16—C17—C14	0.3 (2)	O5—C25—C26—C27	−177.7 (2)
O4—C16—C17—C19	3.2 (3)	C29—C28—C27—C26	0.0 (4)
N1—C16—C17—C19	−175.74 (18)	O6—C28—C27—C26	179.5 (3)
C10—C11—C12—C13	1.7 (3)	C25—C26—C27—C28	0.7 (4)

### Hydrogen-bond geometry (Å, °)

**Table:**

*D*—H···*A*	*D*—H	H···*A*	*D*···*A*	*D*—H···*A*
C4—H4···O4^i^	0.93	2.43	3.294 (3)	155.
C18—H18B···O3^ii^	0.96	2.37	3.325 (3)	171.

Symmetry codes: (i) −*x*+2, −*y*+1, −*z*+1; (ii) −*x*+3, −*y*+1, −*z*+1.
